# Supplementary material for: Transgender Males as Potential Donors for Uterus Transplantation: A Survey
Source: J Clin Med. 2022 Oct 14;11(20):6081. doi: 10.3390/jcm11206081 (PMC9605112; doi:10.3390/jcm11206081)
Supplement: Supplementary file 1 [file jcm-11-06081-s001.zip › jcm-1922454 -Supplementary Material-revise.pdf]

Journal of
Clinical Medicine

MDPI

Supplementary Material

Transgender Males as Potential Donors for Uterus Transplantation: A Survey

Marie Carbonnel [1,2,*], Léa Karpel [1], Ninon Corruble [1], Sophie Legendri [1], Lucile Pencole [1], Bernard Cordier [3], Catherine Racowsky [1,3] and Jean-Marc Ayoubi [1,2]

1 Department of Obstetrics and Gynecology, Foch Hospital, 40 rue Worth, 92150 Suresnes, France
2 Medical School, University of Versailles, Saint-Quentin-en-Yvelines, 55 Avenue de Paris, 78000 Versailles, France
3 Department of Psychiatry, Hospital Foch, 40 Rue Worth, 92150 Suresnes, France
4 Department of Obstetrics, Gynecology and Reproductive Biology, Brigham and Women's Hospital, Boston, MA 02115, USA
* Correspondence: carbonnelmarie@gmail.com

Sir,

You were suffering from Benjamin syndrome. Therefore, you were followed and accompanied in your transition at the Foch Hospital in Suresnes. As part of this transition, you underwent a hystero-oophorectomy in the gynecology department of our hospital. Now, this same gynecology department, which accompanied you on your journey, is proposing to consider the possibility that future patients suffering from Benjamin syndrome not only be offered the possibility of having their uterus removed, but also of being able to donate it, if they so wish, to biological women born without a uterus or having lost it.

In order to evaluate the potential of future donors, we would like to come back to you because we believe that you are the most appropriate people to give your opinion on this possibility. You should know that this questioning is topical because in October 2014, was born in Sweden the first child, carried by a woman whose uterus had been transplanted to her. The child was born healthy as well as his mother. Since then, more than 70 transplants have been done worldwide and 30 babies have been born healthy. The first French birth took place at the Foch Hospital this year. This surgery is currently limited to research protocols. We thought that the current patients suffering from Benjamin syndrome whose uterus would be removed, could in the future be potential donors, in view of a transplantation in women without uterus, having a strong desire for a child and unable to realize it until now. Nowadays, only adoption is accessible to these women because the practice of surrogate motherhood is forbidden in France.

Within the framework of this survey, a statistical processing of your answers will be implemented, in order to realize a scientific communication on the subject. The answers to this questionnaire are not intended to identify you. In order to help us protect your personal data, please do not indicate any identifying information (name, surname, date, etc...) in this survey.

The data in this survey is hosted on a server certified as a health data host.

As the survey is anonymous, no request for access to your data, rectification or deletion can be taken into account after the questionnaire has been completed, as it will be impossible for us to identify you or link you to your survey.

We thank you in advance for taking a few minutes to answer this one.

Pr Ayoubi Jean-Marc and Dr Cordier Bernard

What is your age? ....
Your marital status: Single or In couple
Your family situation:
Are you a parent yourself? Yes/No
children born before transition: Yes/No
Parent by sperm donation: Yes /No
Adoptive parent: Yes /No /Ongoing
Stepfather: Yes/No
As part of your reassignment, your uterus and ovaries were removed and destroyed.
At what age did you have this surgery? ....
Globaly, How did you experience this surgery? Very good, good, bad, very bad, other:.....
On the physical level:
Specify if you had complications Yes/No , if yes describe it .. ..
Specify if you had pain after surgery Yes/No
Psychologically:

Did you sincerely want it? Yes/no
Or was it only to access a change of civil status? Yes/No
Did you experience it as: an amputation, a relief, a mutilation, a right, a forced sterilization, Other….

Uterine transplantation consists of removing the uterus from a voluntary donor and then transplanting it onto a recipient without a uterus. This technique requires a longer operating time (10 hours) than a simple hysterectomy and implies more pre- and post-operative follow-up and therefore more risk of complication: among others, a wound of the ureters (pipe draining the kidney to the bladder), than a simple hysterectomy.

Also, if it had been possible during your reassignment, would you have been willing to donate this organ to benefit a woman born without a uterus or having lost it? Yes/No

If yes, why? Specify……………………………………………………………………………………………………

If no, why not? Specify…………………………………………………………………………………………………

As part of your transition, if you had agreed to be included as a potential donor :

Would you have wanted to know the recipient? Yes/ No

Why?……… ………

Would you have liked to know the outcome of your donation? Yes/No

Why ?……

The following questions would allow us to evaluate the real potential of uterine grafts and call for an evaluation of your gynecological health, prior to the transition.

Before surgery,

Did you have regular gynecological follow-up? Yes/No

Did you have any gynecological pathologies? Yes/No

If yes, which ones? ……………………………………………………

Have you had gynecological surgery? Yes/No

If yes, which one? ………………………………………………

We thank you very much for the time you have devoted to this study and for contributing to great medical projects.
